# Tight Junctions Go Viral!

**DOI:** 10.3390/v7092865

**Published:** 2015-09-23

**Authors:** Jesús M. Torres-Flores, Carlos F. Arias

**Affiliations:** Instituto de Biotecnología, Universidad Nacional Autónoma de México, Avenida Universidad 2001, Colonia Chamilpa, Cuernavaca, Morelos 62210, Mexico; jmtorres@ibt.unam.mx

**Keywords:** tight junctions, virus, JAM-A, occludin, claudins, ZO-1, PDZ

## Abstract

Tight junctions (TJs) are highly specialized membrane domains involved in many important cellular processes such as the regulation of the passage of ions and macromolecules across the paracellular space and the establishment of cell polarity in epithelial cells. Over the past few years there has been increasing evidence that different components of the TJs can be hijacked by viruses in order to complete their infectious cycle. Viruses from at least nine different families of DNA and RNA viruses have been reported to use TJ proteins in their benefit. For example, TJ proteins such as JAM-A or some members of the claudin family of proteins are used by members of the *Reoviridae* family and hepatitis C virus as receptors or co-receptors during their entry into their host cells. Reovirus, in addition, takes advantage of the TJ protein Junction Adhesion Molecule-A (JAM-A) to achieve its hematogenous dissemination. Some other viruses are capable of regulating the expression or the localization of TJ proteins to induce cell transformation or to improve the efficiency of their exit process. This review encompasses the importance of TJs for viral entry, replication, dissemination, and egress, and makes a clear statement of the importance of studying these proteins to gain a better understanding of the replication strategies used by viruses that infect epithelial and/or endothelial cells.

## 1. Introduction

Viruses, as obliged intracellular parasites, need to take advantage of a wide variety of cellular processes to successfully produce infectious progeny. Interestingly, different viruses can exploit the same cellular process, and the biomolecules related to it, in many different ways. In recent years, increasing evidence of the importance of tight junctions (TJs) for the infection of several viruses has arisen, making it clear that studying the role of the components of this cellular pathway during viral replication is important to achieve a better understanding of how viruses make use of the cellular machinery in order to complete their infectious cycle.

## 2. Tight Junction Structure and Function

TJs are highly specialized membrane domains whose main function is to maintain adjacent cells close enough to avoid the free passage of small molecules, microorganisms, and cells across the paracellular space [1,2]. Another important function of TJs is to provide a physical barrier between the apical and basolateral membrane domains of a polarized epithelial cell, thus preventing the free diffusion of membrane molecules from one domain to the other [3].

Besides the maintenance of cell polarity [4,5], TJs are also involved in several signal transduction pathways [6,7], transmitting signals from the outside to the inside of the cell and *vice versa* to regulate several cellular processes like polarization, proliferation, gene expression and differentiation [8,9].

Structurally, TJs are multiprotein complexes formed by several different integral membrane proteins, which are in turn linked to a series of cytoplasmic adaptor proteins that form a scaffold or plaque whose main function is to join the membrane components of the TJ to the actin cytoskeleton and also allow the recruitment of signaling proteins [2,10,11].

**Figure 1:**
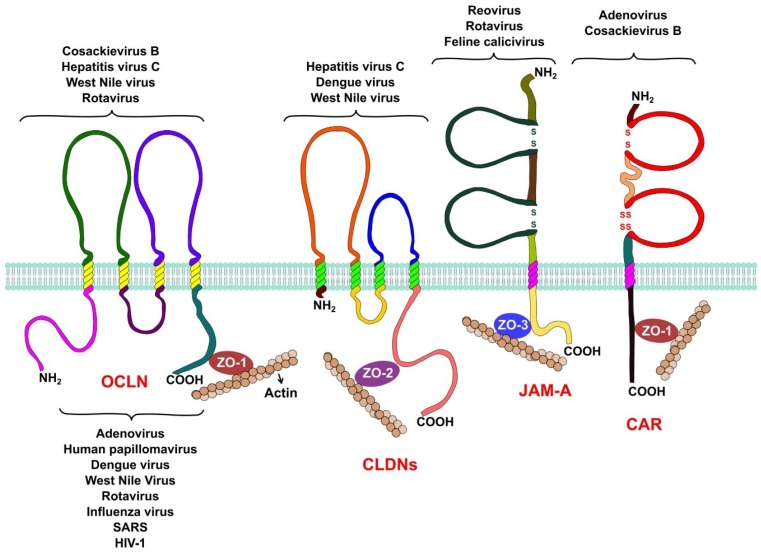
Tight junction proteins and virus replication. Occludin is required by hepatitis C virus, coxsackie B virus, and rotavirus during entry into their host cell. West Nile virus is capable of opening tight junctions by impairing occludin in order to achieve its hematogenous dissemination. Claudins-1, -6, and -9 are used by hepatitis C virus as entry factors. Claudin-1 is also an entry factor for dengue virus, while West Nile virus targets claudin-1 (CLDN-1) to open tight junctions to achieve hematogenous dissemination. Reovirus and feline calicivirus both use JAM-A as a receptor during their entry process, while rotavirus needs JAM-A as a co-receptor. Adenoviruses and coxsackievirus B use CAR as a receptor during their entry. Several proteins of the cytoplasmic plaque that forms tight junctions are involved in viral replication. Rotaviruses require the organization of the plaque provided by the protein zonula occludens protein-1 (ZO-1) for their entry, while other viruses, like influenza, severe acute respiratory syndrome (SARS), and West Nile target this protein to disrupt tight junctions to spread and disseminate. Other plaque proteins, like the multi-PDZ domain protein-1, -2, and -3 (MUPP1, MUPP2, MUPP3), membrane-associated guanylate kinase, WW, and PDZ domain-containing protein-1 (MAGI-1), PALS1-associated TJ protein (PATJ), and zonula occludens protein-2 (ZO-2) are hijacked by adenoviruses and other respiratory viruses, like influenza virus and SARS coronavirus, to open the tight junctions and efficiently exit the airway epithelia. Finally, tight junction proteins are also involved in carcinogenesis, PATJ and MUPP1 are targeted by human papillomaviruses to alter cell polarity, an event that is capable of inducing carcinogenesis in epithelial cells.

The integral membrane proteins that form the TJ belong to three different families (Figure 1). The Junctional Adhesion Molecules (JAM-A, JAM-B and JAM-C) and the Coxsackievirus and Adenovirus Receptor (CAR) belong to the immunoglobulin superfamily. Both proteins span the membrane a single time and have an extracellular domain formed by two Ig-like loops. These proteins also possess a short cytoplasmic tail represented by its C-terminal region, which interacts with other tight junction proteins via PDZ domains [12,13]. Members of this family, like JAM-A and CAR, function as receptors for several viruses.

The claudin family of proteins is another important component of the TJ. Claudins are tetraspaning proteins whose main function is to regulate the passage of ions across the paracellular space [14,15,16]. To date, 27 claudins are known in humans and their differential expression in different epithelia allows the paracellular transport of specific ions in each tissue.

The TJ-associated marvel proteins (TAMPs) are the last transmembrane component of the TJs. This family of proteins has three members, occludin, tricellulin, and marvelD3. A lot of research has been conducted to determine the functions of occludin; even though it is not essential for the formation of TJs [17] this protein is capable of regulating the transport of some small ions across the paracellular space [18]. Occludin as other TAMPs, has a MARVEL domain, which have been shown to associate with cholesterol rich membrane domains and are thought to be involved in the biogenesis of endocytic vesicles and in the organization of specialized membrane domains like the TJs [19]. When the expression of occludin is silenced in epithelial cells by RNA interference, the activation of the Rho GTPase RhoA is impaired under certain stimuli, suggesting that it may also play a role in signal transduction [20]. The roles of both tricellullin and marvelD3 at the TJ are similar to that of occludin but their functions do not overlap [21].

To establish a connection between the outside and the inside of the cell, transmembrane TJ proteins interact with cytoplasmic proteins via the PDZ binding domains (PSD/DlgA/ZO-1) in their cytoplasmic regions. Cytoplasmic proteins like ZO-1, ZO-2, and ZO-3 [22,23], Par-3 and Par-6 [24,25,26], and MAGI proteins [27] also possess PDZ domains and thus readily interact with the transmembrane components of the TJ. The cytoplasmic proteins that form the TJ serve as a scaffold where other signaling proteins such as protein kinase C (PKC), Rho, Rab, and Ras GTPases, and several other proteins that are important for signal transduction from and to the TJ are recruited. PDZ domain containing proteins, such as ZO-1, also play an important role in the assembly of TJs [28].

## 3. Viruses Open the Gate: Tight Junctions during Viral Entry

Many viruses that infect epithelia, like influenza viruses for example, have evolved to use receptors or co-receptors that are readily available on the apical domain of the epithelial cells [29]. On the other hand, many other viruses require basolateral molecules as their attachment and entry receptors [30,31], which are usually inaccessible to viral particles because they are buried under the TJs. To overcome this problem, some viruses have developed elegant strategies to open the TJs in order to reach their receptors, while others are capable of using TJ proteins as receptors.

The first TJ protein that was identified as a viral receptor was the coxsackievirus and adenovirus receptor (CAR). This protein, which belongs to the immunoglobulin superfamily, is usually confined within the TJ and is not readily accessible for viruses to initiate infection. As its name indicates, there are two different viruses known to use CAR as a receptor, adenoviruses and coxsackie B viruses, and they use CAR during their entry in different ways.

Several types of adenoviruses are capable of binding CAR with high affinity through the knob domain of the fiber protein of the virus [32,33]. The binding of adenovirus to the cell surface and its entry into the cell are two separate but highly coordinated events. Once adenovirus binds CAR it is capable of binding its entry receptor; several basolateral molecules such as integrins αvβ3 and αvβ5 [34], α5β1 [35], as well as αvβ1 [36] and α3β1 [37], have all been found to mediate adenovirus entry, clearly showing how important it is for adenoviruses to open the TJs in order to reach these molecules.

In recent years, a splice isoform of CAR (CAR^EX8^) has been associated to the entry of adenoviruses through the apical surface of the airway epithelia [38]. There are two transmembrane splice isoforms of CAR, CAR^EX7^ and CAR^EX8^, which differ only in the last residues of their cytoplasmic domain [39]. This slight difference causes CAR^EX8^ to localize at the apical surface of polarized epithelial cells [38]. IL-8 has been shown to enhance adenovirus entry in a dose-dependent manner, primarily by increasing the expression of CAR^EX8^ at the apical domain [38]. This observation suggests that adenoviruses might take advantage of this host innate immune response, induced by bacteria or other IL-8 stimulating factors, to facilitate infection [38].

Coxsackieviruses, as some adenoviruses that are capable of causing diarrhea (serotypes 40 and 41), enter their host through the oral route, and have to cross the intestinal mucosa in order to reach the bloodstream to initiate viremia and spread to other body sites. To do so, these viruses must gain access to the TJ where they can bind CAR and enter the cell [40].

Not all coxsackievirus strains are able to readily infect the intestinal epithelium, but it has been shown that the strains that are capable of doing so first bind to a protein located in the apical face of the intestinal cells: the decay accelerating factor (DAF) [41,42]. The interaction of viral particles with DAF causes DAF to relocalize into lipid rafts, where it is able to activate the tyrosine kinase c-Abl, which in turn activates the Rho GTPase Rac. The activation of Rac induces a rearrangement of the actin cytoskeleton that facilitates the transport of DAF bound particles into the TJ where the virus can bind CAR and enter the cell [43].

The binding of coxsackie B viruses to CAR induces a series of conformational changes in the viral particle that are critical for the entry and uncoating of the virus. One striking observation is that, as adenoviruses, coxsackieviruses are not endocytosed bound to either CAR or DAF, in fact, these two proteins remain at the cellular membrane while coxackieviruses are internalized [44]. It is worth mentioning that the endocytosis of coxsackie B viruses is coupled with the internalization of another TJ protein, occludin. It is not clear if the viral particles enter the cell together with occludin molecules but they have been shown to co-localize in vesicles inside the cell [44].

Both occludin and coxsackie B virus particles have been shown to enter the cell through different internalization pathways. On the one hand, occludin enters the cell via macropinocytosis, while the viral particles enter the cell through caveolin-mediated endocytosis [44]. Interestingly, the use of drugs that impair the macropinocytosis of occludin also impairs virus internalization; the inverse effect was observed when caveolin-mediated endocytosis of viral particles was blocked. Whether the virus activates the endocytosis of TJs to enter the cell or it promotes the recruitment of caveolin or other regulatory molecules into the vicinity of the TJ still needs to be determined [44]. Interestingly, the entry of echovirus 11 into polarized epithelial cells is dependent on the binding of the virus to DAF. A mutant strain incapable of binding DAF enters the cell through the apical surface, while the wild type strain, after binding DAF, is relocalized to the TJs to enter the cell through the basolateral membrane [45].

A third virus whose receptors lie within the TJ is hepatitis C virus (HCV). Like many other viruses, HCV is able to bind several different molecules on the cell surface, including CD81, SR-BI, and LDLR [46,47]. It was recently demonstrated that the TJ protein claudin-1 is also important for HCV entry even though the virus has not been shown to bind this protein. The localization of claudin-1 in TJs is important for the entry of HCV particles into the cell, since mutations in the first extracellular loop that prevent claudin-1 from establishing cell–cell contacts also impair HCV entry [48]. Claudin-1 has been shown to associate with CD81 to promote virus entry, a property that is also shared with claudin-6 and claudin-9, which have also been found to be important for the internalization of HCV [49]. Another TJ protein associated with the entry of HCV virus is occludin; when this protein is overexpressed in human cells that are not susceptible to HCV infection, the uptake of the virus is highly increased [50]. The step at which occludin participates in the entry of HCV has yet to be determined. Recently, it was demonstrated that dengue virus protein prM/M can also bind claudin-1 and the knockdown of this protein in Huh 7.5 cells prevents the virus from entering them [51].

Rotaviruses are also capable of using TJ proteins for their entry. JAM-A, occludin, and ZO-1 were are all shown to be important for virus internalization [52]. Interestingly, the C-terminal domain of JAM-A seems to be important for the entry of rotaviruses into non-polarized MA104 cells, suggesting that as for coxsackievirus, a signaling pathway associated to a member of this immunoglobulin superfamily is required [52]. The localization of JAM-A in the cell-cell contacts of MA104 cells is also necessary for the entry of rotaviruses. This localization depends on the activity of ZO-1, since when the expression of ZO-1 was silenced using RNA interference, JAM-A was not longer detectable on the cell-cell contacts, and the infectivity of the virus was highly diminished [52]. JAM-A is also a functional receptor for feline calicivirus and there is a clear association between the origin (animal species) of JAM-A and the cellular tropism of the virus [53].

TJs are considered a primary barrier for pathogens due to the fact that they closely seal epithelial cells together so that neither bacteria nor viruses can access the deeper cell layers that lie beneath epithelia. The fact that many viruses have evolved to use TJ proteins as receptors for cell entry may be a way of overcoming their barrier function, using in this way the host defense machinery to their own advantage. The organization of TJs and the proteins that constitute them is specific for each type of epithelium. This might also give viruses that use TJ proteins as receptors some sort of tissue specificity.

## 4. Tight Junctions: Opening the Gates into the Bloodstream

Several studies have clearly demonstrated the importance of JAM-A as a cellular receptor for reovirus [54]. The overexpression of JAM-A in cells not susceptible to reovirus infection makes them capable of supporting viral replication [54]. The viral particles bind JAM-A through the viral protein σ1 with such a high affinity that viral particles are capable of displacing JAM-A/JAM-A interactions [55].

Even though it can be used as a receptor in cultured cells, JAM-A is dispensable for viral replication in the intestine. However, one of the most interesting facts about the association between JAM-A and reovirus *in vivo* involves the hematogenous dissemination of the virus. It was demonstrated that wild-type mice that were orally inoculated with the virus developed central nervous system conditions that were not observed in JAM-A deficient mice (JAM-A −/−) inoculated through the same route [56,57]. When the virus was inoculated directly into the brain, both JAM-A deficient and wild-type mice developed an encephalitis-like condition that suggested that brain cells are susceptible to reovirus infection [58]. These results clearly demonstrated that endothelial JAM-A is necessary for the entry and egress of reovirus into the bloodstream [59].

Several neurotropic viruses have been shown to modify the structure of the endothelial TJs in order to gain access into the bloodstream. West Nile virus is capable of doing so by promoting the degradation of multiple TJ proteins, like JAM-A, ZO-1, occludin, and claudin-1 [60]. A similar effect has been observed during dengue virus infection, where alterations in the actin cytoskeleton, adherens junctions, and a decrease in the expression levels of ZO-1, which alters the integrity of TJs, occurs in endothelial cells [61]. Other viruses, such as human immunodeficiency virus (HIV) and human T leukemia virus (HTLV) have also been shown to disrupt the endothelial TJs to achieve hematogenous dissemination [62,63].

## 5. It Is Not All about the Entry

TJ proteins have been clearly associated to the entry process of several viruses, nevertheless, the cytoplasmic proteins that form the TJ have also been involved in other viral processes. Several membrane and intracellular proteins contain a specialized domain, PDZ, involved in protein-protein interactions and signaling events. Proteins with PDZ domains have been related to the formation and maintenance of TJs and to the establishment of cell polarity, among other functions [64].

In recent years, the role of TJ proteins that contain PDZ domains in viral replication has been established, increasing the evidence of the importance of TJs for the establishment of productive viral infections. Several viral proteins are known to contain PDZ domain-binding motifs or PBMs, but the first evidence of the interactions between PDZ containing proteins and PBMs arose from the study of two oncogenic viruses, adenoviruses and human papillomaviruses (HPV). Adenovirus 9 E4-ORF can bind 4 TJ proteins, MUPP1, MAGI-1, PATJ, and ZO-2 [65,66,67], controlling in this way the formation of TJs. In epithelial cells, the presence of E4-ORF causes the disruption of TJs without causing a disruption of the adherens junction. The disruption of the TJ also causes an impairing of the formation of cell polarity. On the other hand, the HPV E6 protein, which can bind the TJ proteins PATJ and MUPP1, targets them for degradation and causes in this way an alteration of the membrane composition and cell polarity in infected cells. A possible association between the loss of cell polarity and the carcinogenesis induced by HPV has been suggested [68].

Two viruses that cause respiratory illnesses, influenza A viruses and coronaviruses (specifically, SARS coronavirus), have proteins capable of targeting members of the MAGUK family of proteins (MUPP 1, MUPP 2, MUPP 3, or ZO-1) causing in this way the disruption of TJs and alterations in cellular polarity. The functional implications of these changes have yet to be determined, but one possibility is that the disruption of TJs is necessary for the spread of these viruses outside the respiratory tract [69,70].

Infections by herpes simplex virus-1 (HSV-1) and herpes simplex virus-2 (HSV-2) are the most common opportunistic infections in HIV-1 infected patients. The risk of the onset of HSV infections in HIV positive patients has been related to the effect of HIV over the immune system. A recent study has also demonstrated that HIV proteins tat and gp120 are capable of disrupting TJs, facilitating the paracellular spread of both HSV-1 and HSV-2 [71].

The role of several viral proteins with PBM domains has yet to be determined but there is clear evidence of the importance of TJs not only for virus cell entry but also for their spread inside their hosts.

## 6. Modification of TJs for Viral Egress

Viruses are also capable of disrupting the TJs during their exit, in order to successfully egress the epithelia they infect. There are just a few examples of viruses that open the TJ during late steps of their replication cycle. As previously mentioned, the binding of adenovirus particles to their receptor CAR causes a disruption of TJs. This disruption is also exploited by adenoviruses during the late stages of viral replication, since binding of the extracellular adenovirus fiber protein (synthesized *de novo* in infected cells) to CAR causes a disruption of the TJs, allowing the viral particles to exit the respiratory epithelia through the basolateral membrane without getting trapped in the paracellular space [72].

As has been reviewed, the study of TJs is of great relevance to understand the mechanisms used by viruses to successfully infect, replicate, and disseminate within their hosts. Likewise, the characterization of the different stages of viral replication has allowed us to better understand the composition and function of TJs. Table 1 shows a summary of the viruses and the TJ proteins they use to efficiently replicate.

**Table 1:** Summary of the tight junction proteins used by different viruses to efficiently replicate.

	Virus	Target TJ Protein	Function	Reference
DNA viruses	Adenovirus	CAR (CAR^EX7^, CAR^EX8^)	Viral receptor	[32,33,38,39]
		MUPP-1, MAGI-1, PATJ, ZO-2	Impair cell polarity for viral egress and dissemination	[65,66,67,72]
	Human poliomavirus	PAT-1, MUPP1	Alteration of cell polarity, carcinogenesis?	[68]
RNA viruses	Coxsackievirus B	CAR	Viral receptor	[40,43]
		Occludin	Viral internalization	[50]
	Human hepatitis C virus	CLDN-1, CLDN-6, CLDN-9	Viral entry	[48,49]
		Occludin	Viral entry	[50]
	Dengue virus	CLDN-1	Viral entry	[51]
		ZO-1	Alteration of tight junction integrity	[61]
	West Nile virus	JAM-A, ZO-1, Occludin, CLDN-1	Impair tight junctions for hematogenous dissemination	[60]
	Rotavirus	JAM-A, Occludin, ZO-1	Co-receptors	[52]
RNA viruses	Reovirus	JAM-A	Receptor, hematogenous dissemination	[54,55,56,57,58,59]
	Feline calicivirus	JAM-A	Receptor	[53]
	Influenza virus	MUPP1, MUPP2, MUPP3, ZO-1	Alteration of cell polarity, spread outside the respiratory tract?	[69]
	Severe acute respiratory syndrome virus	MUPP1, MUPP2, MUPP3, ZO-1	Alteration of cell polarity, spread outside the respiratory tract?	[70]
	Human immunodeficiency virus 1	Several TJ proteins involved	Disruption of tight junctions, hematogenous dissemination,	[62,63,71]
